# Neutrophil elastase as a diagnostic marker and therapeutic target in colorectal cancers

**DOI:** 10.18632/oncotarget.1631

**Published:** 2014-01-14

**Authors:** Ai-Sheng Ho, Chien-Hsin Chen, Chun-Chia Cheng, Chia-Chi Wang, Hua-Ching Lin, Tsai-Yueh Luo, Gi-Shih Lien, Jungshan Chang

**Affiliations:** ^1^ Division of Gastroenterology, Cheng Hsin General Hospital, Taipei, Taiwan; ^2^ Division of Colorectal Surgery, Department of Surgery, Wan Fang Hospital, Taipei Medical University, Taipei, Taiwan; ^3^ Graduate Institute of Medical Sciences, College of Medicine, Taipei Medical University, Taipei, Taiwan; ^4^ Institute of Nuclear Energy Research, Atomic Energy Council, Taoyuan, Taiwan; ^5^ Division of Hepatology, Taipei Tzu Chi Hospital, Buddhist Tzu Chi Medical Foundation and School of Medicine, Tzu Chi University, Hualien, Taiwan; ^6^ Division of Gastroenterology, Department of Internal medicine, Wan Fang Hospital, Taipei Medical University, Taipei, Taiwan; ^7^ Neuroscience Research Center, Taipei Medical University Hospital, Taipei, Taiwan; ^8^ Research Center for Biomedical Implants and Microsurgery Devices, Taipei Medical University, Taipei, Taiwan

**Keywords:** Biomarker, colorectal cancer, neutrophil elastase

## Abstract

Neutrophil elastase (NE), a serine protease secreted by neutrophils, contributes to the progression of cancers to enhance tumor invasion and metastasis. It has been well reported that the regions surrounding the colorectal cancerous tissues usually are decorated with increased accumulation or aggregation of neutrophils coupled with a higher deposition/expression of NE. Therefore, we hypothesized that an increased expressional level of NE in patients with colorectal cancer (CRC) may represent as one of putative biomarkers for CRC. The aim of this study was to evaluate and assure our hypothesis by measurements of the expressional level of NE in the sera and tissues from CRC patients. Moreover, we also proposed a potential therapeutic strategy by blocking enzymatic activity of NE using sivelestat to inhibit the progression of tumor developments. The infiltrated numbers of neutrophils from specimen tissues of CRC patients, and the secreted forms of NE in the sera were quantitatively measured and compared. To evaluate the serum NE as one of putative biomarkers of CRC patients, the receiver operating characteristic (ROC) curve was made to determine the cut-off value of NE in sera for assurance of CRC diagnosis. To evaluate NE as therapeutic target for CRC, sivelestat, a NE inhibitor, was used and administrated into the CRC xenografts. NE expression level coupled with tumor volume were measured and compared between the control and sivelestat-treated xenografts. We found that more infiltrated neutrophils and an increased NE expression were detected in the cancerous tissues compared to the normal tissues. The serum NE concentration in CRC patients was statistically higher than that in the healthy controls (0.56±0.08 μg/ml vs. 0.22±0.03ug/ml) (*p*<0.05), indicating that serum NE can potentially be a putative marker of CRC. To characterize the role of NE in tumorigenesis, the NE avtivity was detected in HCT-15-xenografts using in vivo imaging system (IVIS). Compare to normal mice, the amounts of active NE in xenografts are significantly higher than normal control animals. In the therapeutic characterizing studies, we found that sivelestat can inhibit tumor growth in the HCT-15-induced xenografts. This study suggests that NE is not only as a putative diagnostic biomarker of CRC, but also a potential therapeutic target for patients suffered with CRC.

## INTRODUCTION

Colorectal cancer (CRC) is one of the most common gastrointestinal cancers with increasing incidence worldwide [1]. Due to the difference in the stage at diagnosis, five-year survival rate of CRC patients varied from 90% to less than 5%. In clinical practice, most patients with CRC were diagnosed in the advanced stage with tumor metastasis resulted in difficulties of treatments, leading to the poor prognosis. Therefore, to uncover reliable early biomarkers from CRC patients is urgent and important. Combined with early detection and treatments, it can prolong the survival rate and improve prognosis of patients with CRC.

The endoscopical and histological verification are considered the gold standard clinical procedure for diagnosis of CRC [2–4]. However, several factors including poor resolution of endoscopy plus sampling error in early small nodules of CRC and inter-personal variations may contribute to fail in correct detections of tumors. Several non-invasive biomarkers for assisting the early detections and diagnosis in gastrointestinal cancers have been reported and used, such as carcinoembryonic antigen (CEA) [5–7]. CRC patients usually express significantly high level of CEA, which may present as a putative diagnostic marker. Moreover, CEA level is also negatively correlated with prognosis. Patients with higher CEA are prone to suffer the recurrence or metastasis of CRC [8–11]. Although increased amounts of CEA is one of markers for early detecting CRC, the diagnostic accuracy (only -50%) using CEA as a maker remain controversial inconclusive because of poor specificity [12]. Therefore, to uncover other CRC-specific serum biomarkers, and/or use a multi-marker cocktail strategy may improve the diagnostic accuracy for CRC [12].

In our previous studies, we found increased numbers of the infiltrated human neutrophils onto the gastric cancerous tissues, suggesting that neutrophils may be positively associated with tumor severity [13]. It has been suggested that initial tumorigenesis is associated with tissue inflammation [14]. In detail, activation and infiltrations of leukocytes including the macrophages and neutrophils causes the entire array of metabolic and physiologic changes during sustained inflammations, leading to tumorigenesis [15]. With unknown reasons, recruited macrophages and neutrophils can somehow shifted to specific subsets of leukocytes such as tumor-associated macrophages (TAM) and tumor-associated neutrophils (TAN), respectively, which initiate or promote tumor formation and progression [16–19]. Therefore, these related altering proteins in responded to the changes of subsets from macrophages or neutrophils to TANs or TAMs may be useful as the surrogate biomarkers of tumors.

It has been suggested that neutrophil elastase is a rational candidate of biomarkers because of increasing expression level of NE during tumorigenesis and its unique bio-effects in promoting tumor proliferation and metastasis [3, 20] [21]. As a matter of fact, neutrophil elastase is a kind of protease which can enzymatically degrade the insulin receptor substrate-1 (IRS-1) resulting in failure to form the association complex of IRS-land phosphoinositide 3-kinase (PI3K), leading to free PI3K in cytoplasm. The free form of PI3K can associate with platelet-derived growth factor receptor (PDGFR) and then induce the tumor proliferative signaling pathway [22]. In summary, NE can act indirectly mediate PI3K-associated downstream signals for tumor cell proliferations. Therefore, this unique characteristic of NE released from neutrophils or TAN is not only as a putative tumor biomarker but also as an important accelerator in tumor progression. In this study, we aimed to measure the expression of NE in serum, and to evaluate NE as a CRC marker for assisting early diagnosis and use a NE inhibitor sivelestat as an alternative therapeutic agent to treat colorectal cancers.

## RESULTS

### Tissue-infiltrated neutrophils accumulated in colorectal cancer

**Figure 1:**
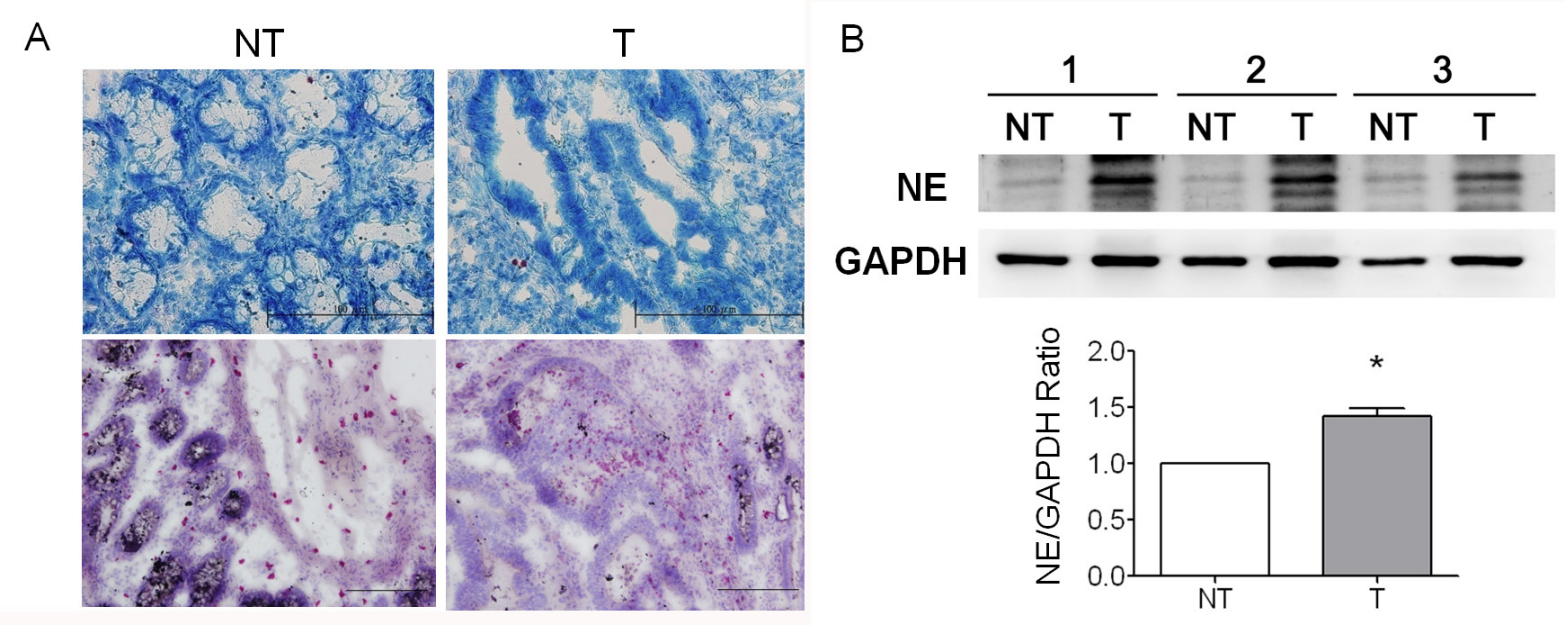
Neutrophils highly accumulated in the tissues of colorectal cancer (CRC) (A) Histopathological confirmation of cancerous tumor (T) and non-tumor (NT) tissues of CRC specimen was figured out using methylene blue staining. Neutrophils were then detected using naphthol AS-D chloroacetate esterase staining. The results indicated that neutrohpils accumulated in the resected tumor (T) tissues of CRC higher than that in adjacent non-tumor (NT) tissues from the same individuals. (B) Western blotting was performed to detect the expression of neutrophil elastase (NE) in T and NT. We found that NE overexpressed in T compared to that in NT *(*p*<0.05*, n=3), indicating that NE secreted by highly accumulated neutrophils can be a tumor biomarker. Scale bar, 200 μm in methylene blue staining and 100μm in neutrophil staining.

Tumorigenesis are always accompanied with constant inflammation in which numerous immune cells such as neutrophils recruit to tumor foci to secret various forms of cytokines and neutrophil elastase (NE), contributing to enhance the tumor progression [23, 24], We have demonstrated that the increased numbers of infiltrated neutrophils in the tissues of gastric cancers were observed in our previous study [13]. To evaluate the role of neutrophil infiltrations and NE in the tumorigenesis/progression of colorectal cancer, we first quantified the numbers of infiltrated neutrophils onto CRC and then measured the NE expression level. The numbers of infiltrated neutrophil and the expressional level and activity of NE derived either from regions of cancerous tissue (T) or the paired non-tumor region (NT) were measured and compared. To differentiate cancerous tissue (T) and the paired non-tumor region (NT), the CRC specimens were stained with methylene blue (Figure 1A). To measure the numbers of the infiltrated neutrophil onto CRC tumor region, the naphthol AS-D (3-Hydroxy -2-naphthoic-o-toluidide) chloroacetate, a substrate specific for neutrophil esterase, was used. The results indicated that the numbers of the infiltrated neutrophils were significantly increased in the CRC tumor tissues as compared to non-tumor region shown in Figure 1A. This result was consistent to our previous studies on the gastric cancers. Furthermore, we also observed the tumor tissues from CRC xenografts with increased infiltrated neutrophil numbers as seen in clinical specimens (data not shown).

We further evaluated and compared the expression level of NE between non-tumor (NT) and CRC tumor regions (T) using western blotting. The results indicated that higher expression level of NE in tumors compared to that in non-tumor regions as shown in Figure 1B, demonstrating that NE expression was significantly increased in CRC tumor tissues and may present as a candidate of CRC biomarkers.

### Neutrophil elastase expressed in sera of CRC patients as a diagnostic marker

**Figure 2:**
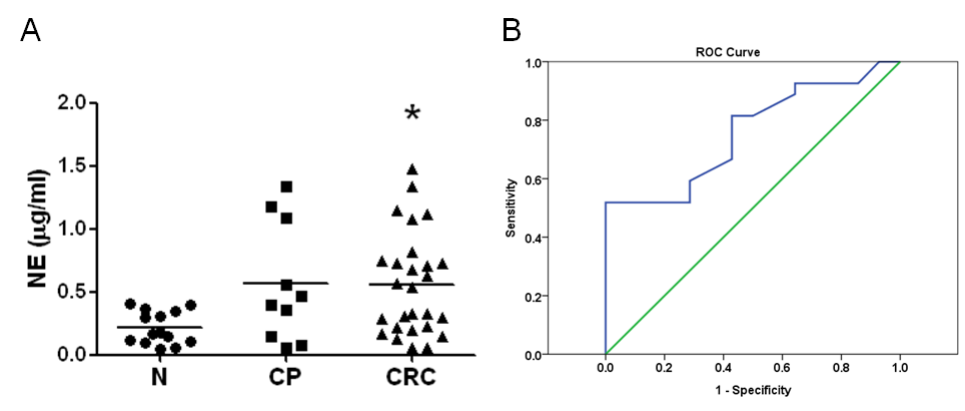
Elevated NE level in the sera of colorectal cancer patients (A) The sera from three groups including healthy volunteers (n=14), colorectal polyp (CP, n=10), and colorectal cancer (CRC, n=27) were enrolled and compared. NE was measured as 0.22±0.035μg/ml in healthy volunteers, 0.57±0.15μg/ml in CP, and 0.56±0.078μg/ml in CRC (*p*<0.05 compared to healthy group). The result indicated that NE was a reliable marker for predicting CRC. (B) The ROC data suggests that the sensitivity and specificity of NE were 0.63 and 0.643, respectively, with 0.31μg/ml of cut-off value.

Neutrophil elastase (NE) is a protease which can cause damage and then generate favorable environments for carcinogens tumor progression. Furthermore, NE can enzymatically degrade insulin receptor substrate-1 (IRS-1) and then increase the interactions of phosphatidylinositol 3-kinase (PI3K) and the potent mitogen platelet-derived growth factor receptor (PDGF), triggering to tumor cell proliferation [22]. Therefore, tumor-deposited NE secreted from neutrophils may be useful as a candidate biomarker for benign and malignant diseases [25, 26]. To evaluate the role of NE in CRC diagnosis, we measured and compared the amounts of NE from three different sources of serum including healthy volunteer, individuals with colon polyp (CP), and patients suffered with CRC. The results indicated that the NE concentration was significantly higher in the CRC specimens than that in healthy controls (Figure 2A, 0.56±0.08 μg/mL *vs*. 0.22±0.03 μg/mL, *p*<0.05), indicating that NE could be a serological marker of CRC. Moreover, the sensitivity and specificity of NE were 0.63 and 0.643, respectively, with a cut-off value of 0.3 1μg/mL according to ROC analysis (Figure 2B).

### The higher activity of neutrophil elastase (NE) in CRC tumor could be inhibited by sivelestat, indicating NE as a useful target of CRC

**Figure 3:**
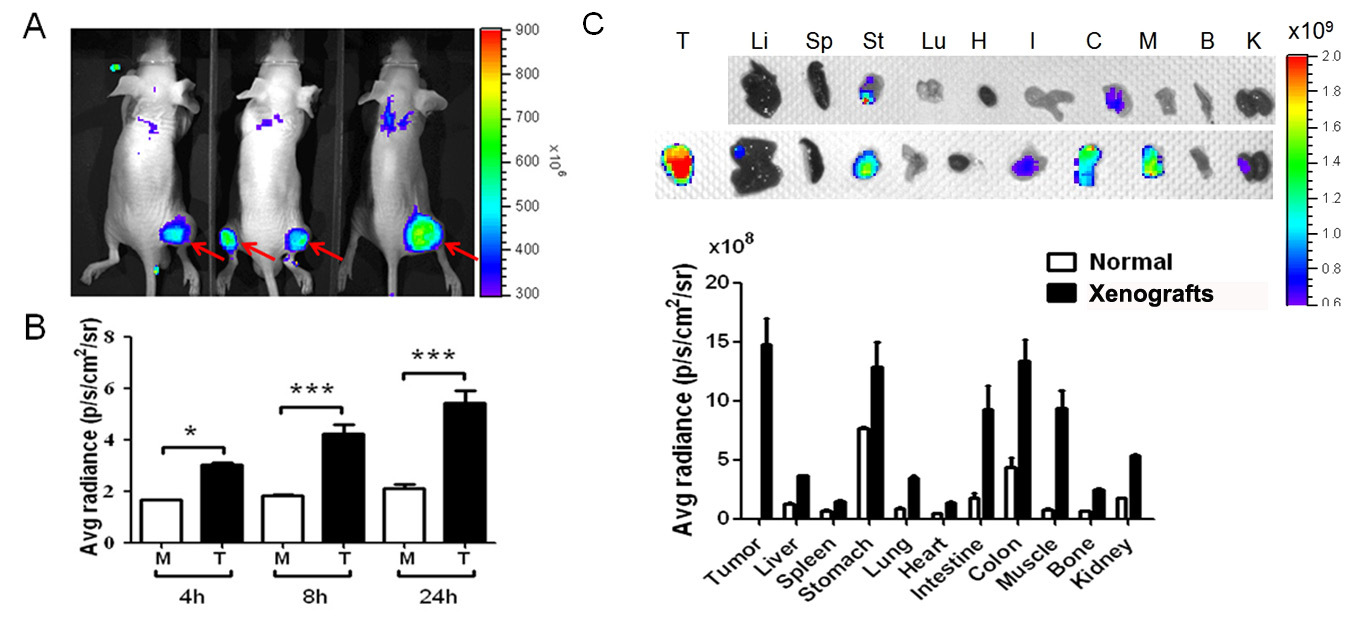
The neutrophil elastase (NE) activity was measured in CRC xenografts using an in vivo imaging agent based on a peptide (amino sequence, PMAWQSVP) which was specifically recognized by NE (A … B) NE activity was gradually elevated from 4h to 24h in tumors (T) compared to muscle (M) after the agent administration via tail vein injection, indicating that NE can be a reliable theranostic target for CRC. (C) Biodistribution assay of NE expression in the each organ between normal nude mice, without HCT-15 implatation, and CRC xenografts showed that NE activity was highest in tumors compared to other organs in CRC xenografts, and HCT-15 implantation caused higher NE expression compared to normal mice in all organs. *p<0.05, **p<0.01, ***p<0.001.

The NE level in serum from CRC patients was statistically elevated as indicated in Figure 2. Therefore, we would like to clarify the correlations between NE and tumors. In figure 3A, we demonstrated that NE enzymatic activity is strongly associated with tumors. This experiment was performed by intravenously injection of the Neutrophil Elastase 680 FAST imaging agent into HCT-15-induced xenografts, and then we measured the fluorescent intensity in three different time point at 4, 8 and 24 hours. The real time imaging results demonstrated that tumor regions displayed the most strongest fluorescent intensity, indicating around tumor foci with the maximal NE enzymatic activity (Fig 3B, *p*<0.05). Furthermore, to detail and measure the amounts of NE in various organs and tissues, the biophysical -distribution assay was performed. Beside stomach and colon with high NE activities, the limited NE enzymatic activities were detected in the most organs including liver, spleen, lung, heart and bone from mice without implantation of HTC-15 tumors (Fig 3C). In the xenografts, however, the strongest enzymatic activity was noted in the tumor region. Again, we demonstrated that xenografts displayed with significantly elevated NE activities in tumors, indicating that NE can be a putative candidate of CRC makers.

**Figure 4:**
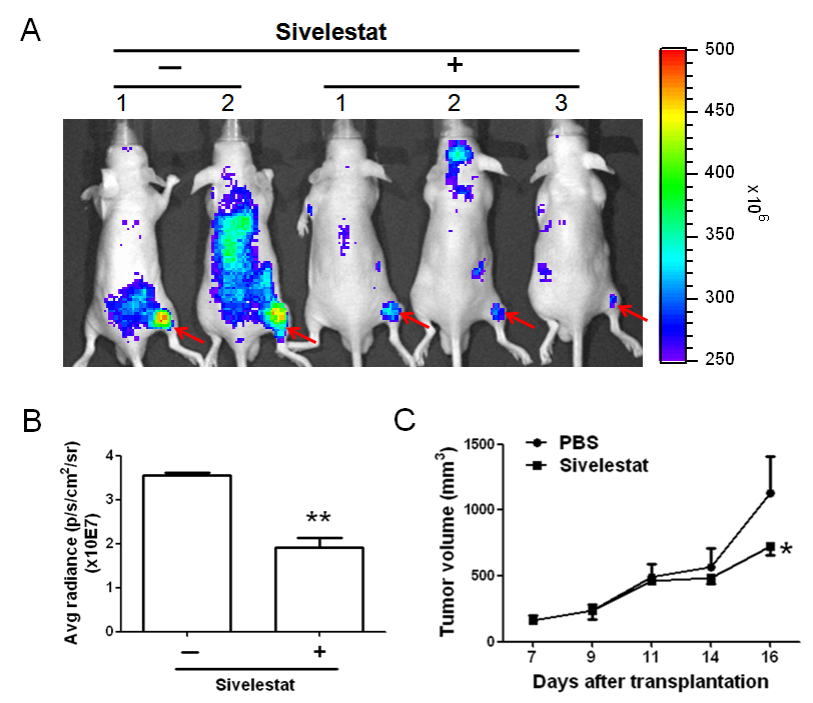
NE was a CRC therapeutic target (A … B) The neutrophil eleatase inhibitor, sivelestat, was performed to suppress NE activity. The inhibitory effect of NE activity was measured using real-time in vivo imaging system (IVIS), which indicated that sivelestat can reduce the NE activity in tumor. (C) Sivelestat can furthermore reduce the tumor growth in vivo (*p<0.05), demonstrating that NE was a CRC therapeutic target. **p<0.01.

We revealed the associations of NE and tumors. Furthermore, previous studies suggested that NE can induce tumor cells proliferation [22]. Taking together, we hypothesized that tumor growth and progression may be inhibited or limited by reduced NE activity using NE-specific inhibitors. To examine our hypothesis, sivelestat, a NE specific inhibitor, was used. We first performed *in vitro* cytotoxicity analysis by culturing HTC-15 cells in the medium containing sivelestat at concentrations of 1, 10, 50 or 100 μg/ml. The results showed sivelestat at concentration greater than 50 μg/ml directly inhibited HTC-15 cell proliferation (data not shown). Followed by *in vitro* cytotoxicity assay of sivelestat, we evaluated the inhibitory effects on enzymatic activity by intravenously injection of 300 ug sivelestat combined with NE imaging agent to each CRC xenograft. The results shown in Figure 4A and 4B demonstrated that sivelestat can significantly inhibit *in vivo* NE activity, especially in the adjacent regions of tumors. To investigate the therapeutic efficacy of sivelestat, 100μg of sivelestat was injected to each CRC xenografts every other day for 3 times. The results demonstrated that the tumor volume in the xenografts with injection of sivelestat was statistically diminished near to two fold reduction (Figure 4C, *p*<0.05), compared to that in animals with only PBS injection at day 16 after tumor transplantation, indicating that targeting to NE activity may be a potential or alternative therapeutic approach to eradicate or limit CRC tumor growth and progression.

## DISCUSSION

In this study, we evaluated and demonstrated NE in serum as a putative biomarker for the diagnosis of CRC, and blocking NE activity using sivelestat or other NE inhibitors/blockers may provide a new line of therapeutic drugs to limit CRC progression. Beside increased NE level in the serum of CRC patients, we also found tumor tissues with a higher NE expression in compared with that in the paired non-tumor regions in enrolled individuals. Moreover, NE level was higher in sera of CRC patients compared to healthy volunteers. The results indicated that NE was a reliable and useful diagnostic biomarker of CRC. On the other hand, a NE inhibitor, sivelestat, was used to impair NE enzymatic protease activity and evaluate its therapeutic effects by measuring and comparing the tumor volume between HCT-15 xenografts with or without administrations of sivelestat. The results revealed that sivelestat significantly reduced tumor growth, suggesting that sivelestat was a good CRC therapeutic candidate and NE may be a therapeutic target for CRC.

Previous studies have demonstrated that neutrophils participate in the development and metastasis of tumor [15, 22, 27–29], thus suppression of neutrophils is also a proposed strategy for tumor therapy. Overproduction of NE in tumor tissue elucidates character of neutrophils on promoting tumor growth. Literatures also report that NE secreted from neutrophils is a prognostic marker [25, 26], and can trigger tumor proliferation via degrading IRS-1 in tumor cells [22]. Therefore, NE may be a target for anti-tumor growth. In this study, we validated the overexpression of NE in tumor tissues and sera of CRC, demonstrating that NE was a reliable biomarker of diagnosing CRC.

Under normal physiological condition, the activity of secreted NE from neutrophils is regulated by protease inhibitor such as alpha-1 anti-trypsin (A1AT). However, in tumor circumstance, A1AT is down-regulated in tumor tissues [30]. The imbalance between NE and anti-protease also enhances the NE-induced tumor development. Except degradation of IRS-1, NE also cleaves pro-TGF-α to release mature TGF-α in gastric TMK-1 cells, which activates EGFR-induced tumor proliferation [31, 32]. Based on these evidences, blockage of NE activity is a good rational therapeutic target. The anti-NE agents, such as sivelestat, can be a possible candidate or adjuvant for fighting tumors.

Basically, NE secreted by neutrophils was proposed to enter blood as a serum biomarker. Therefore, the serum NE levels were measured (Fig 2A). In the enrolled CRC patients and healthy volunteers, the sensitivity and specificity of NE were measured as 0.63 and 0.643, respectively, with cut-off value 0.31μg/ml. We also measured the CEA levels, in the enrolled individuals. The results indicated that the sensitivity and specificity of CEA applied in clinical practice are 0.593 and 0.643, respectively, with the cut-off value of 2 ng/ml (data not shown). Compared to CEA, NE had a similar diagnostic accuracy. Besides, there was no correlation between NE and CEA, therefore, we suggest that combination of NE with CEA may benefit the diagnostic accuracy for CRC.

Except the identification of NE as a reliable biomarker of CRC, we also demonstrated the highly accumulation of neutrophils in tumor foci. This character of neutrophils may also be a target for detecting tumors. For example, a neutrophil targeting SPECT imaging agent cFLFLF-PEG-TKPPR-Tc-99m is used to detect activated neutrophils in the site of inflammation [33], which may also predict prognosis of tumor and help evaluating therapeutic effect. But while using NE as a marker to predict CRC, other diseases involved in neutrophils activation such as acute lung injury should be excluded.

In conclusion, NE was overexpressed in the sera and tissues of CRC patients as a diagnostic and a therapeutic biomarker. In this study, NE levels were measured in 14 healthy volunteers and 27 CRC patients. The results revealed that the cut-off value of NE was 0.31 μg/ml for distinguishing CRC from health. Thus, NE could be used as a diagnostic biomarker for CRC in clinical practice. Regarding NE as a therapeutic target of CRC, NE inhibitor such as sivelestat could be a candidate applied for the treatment of CRC in the future.

## METHODS

### Acquisition of the tissues of colorectal cancer

The study of acquisition of the clinical samples was approved by the Institutional Review Board of Cheng Hsin General Hospital (CHGH-IRB-(240) 100-01). The pairs of tissues including tumors (T) and adjacent non-tumors (NT) from the individuals of CRC were captured followed by surgery. The stages of the tumor were determined by a pathologist using histological staining examination according to the rules of American Joint Commission on Cancer Staging (AJCCS) system. The sera were also collected from patients with CRC or healthy volunteers.

### Neutrophils detection and neutrophil elastase imaging

The pairs of 10 μm-thick tissues from CRC patients cut by a cryostat (HM525, Thermo Scientific Microm, Germany) at -20 °C were attached on the general glass slides for methylene blue staining and for naphthol AS-D (3-Hydroxy-2-naphthoic-o-toluidide) chloroacetate esterase staining. In methylene blue staining, the slides were fixed using 37% of formadehyde for 15 mins. The fixed tissues were then immersed in 0.1% of methylene blue for 1 min after PBS buffer (10 mM sodium phosphate, pH7.4 and 0.9% sodium chloride) washing, and consequently washed using tap water. For neutrophils staining, naphthol AS-D (3-Hydroxy-2-naphthoic-o-toluidide) chloroacetate esterase staining method (Sigma, USA) was performed.

Moreover, the Neutrophil Elastase 680 FAST imaging agent (PerkinElmer, USA) was performed to detect NE in vivo. The NE imaging agent is an activated agent that is optically silenced, but can produce fluorescent signal after cleavage by neutrophil elastase [34]. The half-life of Neutrophil Elastase 680 FAST imaging agent in plasma is 4 hours. After 4 hours of intravenous injection of the dose of 4 nmols (100 μL) into CRC xenografts via tail vein, in vivo imaging system (IVIS, PerkinElmer, USA) was performed to capture the imaging for detecting NE activity. The fluorescent intensity of NE in tumor was compared to that in muscle according to the student *t* test.

### Western blotting

The pairs of tissue samples were homogenized (Pro 200, Bertec, USA) in the lysis buffer (10 mM of sodium phosphate, 0.9 % of sodium chloride, 1% of triton-X100, pH7.4). After getting rid of the precipitated pellets by centrifugation (10000 rpm, 5 minutes), the peppetted supernatants were added with the sample buffer (10 mM of sodium phosphate, 0.9 % of sodium chloride, 8 M of urea, 30 % of glycerol, 2 % of sodium dodecyl sulfate, 0.1 % of β-mercaptoethanol and 0.1% of bromophenol blue) by 1: 1 ratio, and boiled at 100 °C for 5 min for protein denature. Approximately 20 μg of each sample protein was loaded onto the individual grid of 4-12 % sodium dodecyl sulfate-polyacrylamide gel electrophoresis (SDS-PAGE, Invitrogen, USA). The iblot dry blotting system (Invitrogen, USA) was used to transform the proteins to polyvinylidene fluoride (PVDF) membrane based on ion flowing along with a copper electrode. After using 0.5 % of milk to blot the PVDF membrane for 30 min, the individual 2 μg/ml of primary antibody was added for incubation for 2 hour on shaking. The consistent secondary antibodies conjugated with 2 μg/ml of horseradish peroxidase (HRP) were incubated for 1 hour on shaking at room temperature. Between the incubating processes, three-times washing by PBS buffer (10 mM of sodium phosphate, pH7.4 and 0.9% of sodium chloride) were necessary. The ECL detection system (Merck Millipore, USA) was performed, and the images were acquired by Imaging System (Gel Doc XR System, Bio-Rad, USA) depending on the moderate exploring time.

### Tumor inhibition assay

Tumor xenografts were established by injecting 2 × 10^6^ HCT-15 cells into the subcutaneous legs of nude mice aged 6-8 weeks. The tumor imaging and therapeutic experiments were performed after 2 weeks from tumor cells injection. First, the inhibitory effect of sivelestat to NE activity was investigated using real-time IVIS technique. The Neutrophil Elastase 680 FAST imaging agent (PerkinElmer, USA) was co-injected with 300 μg of sivelestat. In addition, 100 μg of sivelestat was injected via tail vein for 3 times in tumor therapeutic assay, whereas PBS buffer was used as control. The tumor volume was calculated as the following formula: length × width^2^ × 0.52.

### Statistic analysis

The statistic software GraphPad Prism 5 (GraphPad Software, Inc. USA) was performed to calculate the differential significance using Bonferroni's Multiple Comparison Test. The ROC curve was completed using SPSS software. The significance difference *(p* value) was acceptable as < 0.05.

### COMPETING INTERESTS

The authors declare that they have no competing interests.
